# Presence of age- and sex-related differences in heart rate variability despite the maintenance of a suitable level of accelerometer-based physical activity

**DOI:** 10.1590/1414-431X20198088

**Published:** 2019-08-05

**Authors:** G.D. Spina, B.B. Gonze, A.C.B. Barbosa, E.F. Sperandio, V.Z. Dourado

**Affiliations:** Laboratório de Epidemiologia e Movimento Humano, Universidade Federal de São Paulo, Santos, SP, Brasil

**Keywords:** Autonomic balance, HRV, Aging

## Abstract

There is currently a lack of information adjacent on the influence of sex and age in heart rate variability (HRV), adjusted according to accelerometer-based physical activity (PADL). We hypothesized that the effect of sex and age on the HRV should be reduced or absent in individuals with a suitable PADL level. We aim to evaluate the influence of sex and age on HRV, adjusted for the confounding effects of the PADL level. A total of 485 age-stratified subjects (18–39, 40–59, and ≥60 years) underwent HRV analyses at rest and 7-day assessments of accelerometer-based PADL. Multivariate analyses of covariance were done using log-transformed HRV indices as outcomes, age and sex as fixed factors, and PADL, cardiovascular risk, fat body mass, and heart rate (HR) at rest as covariates. Despite the adjustment for directly measured PADL, women had better indices of vagal tone, whereas men had higher sympathetic influence. Also, compared to middle-aged and older adults, younger individuals (ages 18–39 years) presented better HRV. Multiple regression analyses confirmed that age and sex were the main predictors of HRV indices, even after adjusting for PADL directly assessed by triaxial accelerometer and HR. We also observed that the correlation between some HRV indexes and the different indexes of physical activity directly evaluated was significant, but not very consistent. Thus, HRV indices are influenced by age and sex, regardless of accelerometer-based physical activity. Interventions with physical activity and exercise aimed at improving the autonomic modulation of asymptomatic adults should take such differences into account.

## Introduction

Heart rate variability (HRV) analysis is a non-invasive method of evaluating the variations in the autonomic system between heart beats through a temporal and spectral analysis of the exponent of the RR intervals. The association between HRV and cardiovascular disease (CVD) is well established in the current literature. Studies have shown that there is an increase in all-cause mortality and morbidity in cases with an abnormal increase in sympathetic or decrease in parasympathetic activity of the cardiac muscle (1).

Sex and age are determinants of autonomic modulation and can hence affect the HRV (2). Some studies have suggested that women exhibit parasympathetic dominance and a decrease in sympathetic activity in terms of heart rate control compared to men of the same age; such an association implies a greater cardiovascular advantage for women (3,4).

The level of physical activity in daily life (PADL) also influences HRV through an increase in vagal tonus (3). However, the studies assessing this topic have primarily included face-to-face interviews or administered PADL questionnaires (5). Such questionnaires have been extensively used due to their applicability in large sample sizes, low cost, and the ability to obtain detailed information regarding the type of physical activity and its context; however, the trustworthiness of the answers is a known limitation of these instruments (6). In contrast, triaxial accelerometers provide an accurate measurement of the PADL by precisely identifying instances where subjects fail to perform the recommended 30 min of moderate to intense physical activity a day, for 5 days a week (7).

Although several studies have analyzed the influence of sex and age on the HRV, scientific knowledge about this influence of HRV, when adjusted for objectively measured PADL, is lacking. In the present study, we aimed to evaluate the influence of sex and age on the HRV of adults, while adjusting for the confounding effects of PADL that was measured using triaxial accelerometers. In addition, we sought to assess the cardiovascular risk factors associated with HRV.

## Material and Methods

### Subjects

We enrolled 485 individuals aged between 18 and 80 years who participated in the Epidemiology and Human Movement Study (EPIMOV). The EPIMOV study is a prospective cohort study that primarily aims to investigate the longitudinal association between sedentary behavior and physical inactivity, and the occurrence of hypokinetic diseases, particularly cardio-respiratory diseases. Volunteers were recruited through outreach programs on social networks, as well as via notices displayed in universities, local magazines, and newspapers. Participants who reported a previous medical diagnosis of heart disease, lung disease, and/or musculoskeletal problems were excluded. Participants who were taking beta-blockers were also excluded. After selection, the subjects were informed about the procedures to be performed, and all the participants provided written informed consent. The project was approved by the Ethics Committee for Research on Human Beings of the local university (ECR: 186.796).

Prior to the evaluations, volunteers answered questions on previous health problems, medication use, and risk factors for CVD, including age, family history, smoking, hypertension, dyslipidemia, diabetes, and obesity. Body weight and height were measured according to the recommended method (8), and the body mass index (BMI) was calculated by the formula: weight/height^2^ (kg/m^2^).

### Assessment of HRV

The RR intervals were recorded at rest using a heart rate monitor (RS800, Polar Electro, Finland) in order to assess HRV as previously described (9). Volunteers were monitored for 10 min in the supine position; during that period, they were asked to breathe normally and to not talk, sleep, or move (2). Prior to the assessment day, the volunteers were instructed to fast and specifically avoid the intake of coffee, tea, soft drinks, and alcoholic beverages. Moreover, the subjects were asked not to smoke or participate in physical activity prior to the evaluation. The heart rate sensor was fixed to the chest, and measurements were initiated when the signal had stabilized (2).

After monitoring, the data were transferred to a computer and stored using the Polar ProTrainer 5 software (Polar Electro). An intermediate 5-min window was selected and its quality was visually assessed regarding the stationary periods; if necessary, another 5-min interval was adopted (2). If an RR interval differed from its adjacent intervals on average more than a specific threshold value, the interval was identified as an artifact and was marked for correction. We have chosen the medium artifact correction, which means that any RR interval that was larger/smaller than 0.25 s compared to the locale average was corrected by replacing the identified artefact with interpolated values using a cubic spline interpolation. We adjusted this threshold with mean heart rate. A good quality interval was defined as the lack of any large outliers of RR intervals, an equal distance between consecutive points of RR measurement, and the presence of minimum variation (2). Each data point was stored and transferred to the Kubios HRV software (University of Eastern Finland).

To analyze the mean time-domain of the normal RR values (mean RR), the standard deviation of the normal RR (SDRR), square root of the sum of the successive differences between normal adjacent RR squared (RMSSD), and percentage difference in RR with an adjacent data point after >50 ms (pNN50) were measured.

In the frequency domain, we evaluated the power of the high- (HF, 0.15–0.40 Hz), low- (LF, 0.04–0.15 Hz), and very low- (VLF, 0–0.04 Hz) frequency bands. We report HF, LF, and VLF in absolute and normalized values. We used the fast Fourier transform (FFT) in which the RR intervals were resampled in the time (4 Hz), and the frequency spectrum was divided into 1,024 points (1). We also calculated the LF/HF ratio.

By using the Poincaré plot (10), we quantified the instantaneous variability of the RR intervals (SD1) and the long-term variability (SD2), and assessed the relationship between these 2 indices. Moreover, we estimated the geometric indices Alpha 1 and Alpha 2.

To control for the influence of the respiratory rate in the analysis, we excluded data from the first 2.5 min of the test; thereafter, the participants were assumed to have resumed a normal breathing pattern.

### Assessment of PADL level

Triaxial accelerometers (GT3X+, ACTIGRAPH, USA) were used to measure the PADL level. The device accurately records the intensity of physical activity, stratified into sedentary lifestyle, light physical activity, moderate physical activity, vigorous physical activity, and very vigorous physical activity. Each participant received the following instructions regarding accelerometer use: the device should be attached with the band on the dominant side, just above the hip, for at least 10 h over 7 consecutive days. The volunteers were also asked to not wear the accelerometer while sleeping, showering, or performing any aquatic activity. The minimum level of physical activity in terms of quantity and intensity was considered to include 150 min per week of moderate to vigorous physical activity (MVPA) (7). Individuals who had not achieved this level of physical activity were considered physically inactive.

### Statistical analysis

We first performed descriptive data analysis, including the generation of frequency histograms and standard error charts. The level of PADL was assessed as a dichotomous variable (i.e., presence or absence of physical inactivity) and as a continuous variable (e.g., step count of the accelerometer, as well as the quantity and intensity of the physical activity). The correlations between continuous variables were assessed using Pearson or Spearman correlation coefficients as a symmetric or asymmetric distribution of the variables, respectively. The proportions were compared using the chi-squared test.

Participants were stratified into the following age groups: 18–39 years, 40–59 years, and ≥60 years. To evaluate the influence of sex and age on HRV, we performed logarithmic transformation of the data so that we could compare the three age groups according to sex in multivariate analyses of covariance (ANCOVA). In these models, we considered the HRV indices as the outcome and age groups and sex as the fixed factors. We checked the interaction between age and sex. Models were adjusted for the main covariates, i.e., MVPA, cardiovascular risk, and body composition (fat body mass). Since using 5-min windows can lead to a different window-length in terms of beats, and differences in heart rate can be very likely related to age and gender, we also adjusted our ANCOVA models for HR at rest during the 5-min window. Post-hoc comparisons and Bonferroni correction were used to identify between-group differences. Also, to increase the statistical power of the multivariate models, we calculated the Framingham cardiovascular risk score (CVRS), including age, sex, body mass index, hypertension and its treatment, diabetes, and smoking (11). We did a sensitivity analysis and, due to the strength of the correlation, we chose the CVRS as a covariate instead of inserting the cardiovascular risk factors. The CVRS was calculated as a percentage as recommended (11). The purpose was to identify the HRV indices that were influenced by age and sex, despite the influence of other variables, mainly PADL. Thereafter, stepwise linear multiple regression models were developed using the logarithm-transformed HRV indices as outcomes. Age as continuous measure, sex, and MVPA in min/week were the main predictors included in the models, and were adjusted for the main confounders, including CVD risk and fat body mass. The probability of an alpha error was set at 5%.

## Results

Of the 485 patients, we primarily enrolled women (n=304) aged 40–59 years. Most of the women were eutrophic, whereas most of the men were overweight. The risk of CVD increased with increasing age (Supplementary Table S1).

Regardless of the assessed age, women had higher indices of vagal tone (e.g., HF values), whereas men had higher sympathetic activity. Moreover, men had higher LF/HF values at all ages. In addition, men and women aged 18–39 years had better HRV indices than those of men and women aged 40–59 years and >60 years, regardless of sex (Supplementary Table S2). We observed significant correlations between the intensity of PADL and some HRV indices (Table 1).

**Table 1:** Results of multiple regression analyzes with the influence of age and sex as predictors of indices of heart rate variability.

Outcome	Main predictors	ΔR^2^	P value	Total R^2^
Ln-SDRR (ms)^*^	Age (years)	0.221	0.000	0.407
Ln-RMSSD (ms)	Age (years)	0.180	0.000	0.476
	Sex	0.004	0.025	
Ln-NN50 (ms)^*^	Age (years)	0.166	0.000	0.306
	Sex	0.008	0.014	
Ln-pNN50 (ms)^*^	Age (years)	0.135	0.000	0.332
	Sex	0.006	0.023	
Ln-HF (ms^2^)^*^	Age (years)	0.204	0.000	0.488
	Sex	0.014	0.000	
Ln-HF (%)	Sex	0.039	0.000	0.185
	Age (years)	0.039	0.000	
Ln-HF (n.u.)	Sex	0.056	0.000	0.185
	Age (years)	0.018	0.000	
Ln-LF (ms^2^)	Age (years)	0.242	0.000	0.376
Ln-LF (%)	Sex	0.020	0.000	0.048
Ln-LF (n.u.)	Sex	0.055	0.000	0.155
	Age (years)	0.010	0.005	
Ln-LF/HF	Sex	0.046	0.000	0.096
Ln-SD1 (ms)^*^	Age (years)	0.189	0.000	0.522
	Sex	0.009	0.000	
Ln-SD2 (ms)^*^	Age (years)	0.227	0.000	0.393
Ln-SD1/SD2	Sex	0.032	0.000	0.278
	Age (years)	0.031	0.000	
Ln-Alpha1	Sex	0.051	0.000	0.209
	Age (years)	0.023	0.000	
Ln-Alpha2	Age (years)	0.038	0.000	0.089

Models adjusted for age, sex (males=1; females=0), accelerometer-based moderate-to-vigorous physical activity (MVPA, min/week), Framingham cardiovascular risk score (%), and fat body mass (%). *MVPA selected as a significant predictor. Ln: logarithm transformation; SDRR: standard deviation of normal RR intervals; RMSSD: root mean square of the RR intervals; pNN50: percentage of normal RR intervals that differ more than 50ms from its adjacent; HF: high-frequency power; LF: low-frequency power; SD1: instantaneous variability of RR intervals; SD2: long-term variability; alpha 1 and 2: detrended fluctuation indices; n.u.: normalized units.

Multiple regression analysis indicated that age, sex, and physical inactivity were the main predictors of the HRV indices. Accordingly, age was found to be an independent predictor of 14 out of 16 HRV indices assessed. Moreover, sex was found to be a determinant of the 12 HRV indices. The regression models explained between 8 and 18.9% of the total variability in the HRV indices (Table 2).

**Table 2:** Correlation coefficients of the indices of the heart rate variability (HRV) and physical activity.

	Physical activity intensity (min/week)					
	Sedentary	Light	Moderate	Vigorous	Very vigorous	Moderate-to-vigorous
SDRR (ms)	0.019	-0.075^*^	-0.075^*^	0.358^**^	0.291^**^	0.255^**^
P value	0.587	0.028	<0.001	<0.001	<0.001	<0.001
RMSSD (ms)	-0.033	-0.007^*^	0.156^**^	0.299^**^	0.258^**^	0.229^**^
P value	0.343	0.026	<0.001	<0.001	<0.001	<0.001
pNN50 (% total)	-0.028	-0.076^*^	0.152^**^	0.302^**^	0.253^**^	0.224^**^
P value	0.421	0.028	<0.001	<0.001	<0.001	<0.001
HF (ms^2^)	-0.007	-0.052	0.167^**^	0.296^**^	0.247^**^	0.198^**^
P value	0.840	0.127	<0.001	<0.001	<0.001	<0.001
HF (%)	-0.007	-0.038	0.009	0.074^*^	0.072^*^	0.019
P value	0.831	0.272	0.792	0.031	0.036	0.583
HF (n.u.)	-0.017	-0.039	-0.009	0.042	0.053	-0.001
P value	0.626	0.260	0.801	0.220	0.120	0.976
LF (ms^2^)	0.014	-0.040	0.194^**^	0.323^**^	0.247^**^	0.229^**^
P value	0.680	0.247	<0.001	<0.001	<0.001	<0.001
LF (%)	0.012	0.047	0.034	0.026	-0.017	0.033
P value	0.731	0.176	0.317	0.451	0.627	0.331
LF (n.u.)	0.003	0.033	0.005	-0.042	-0.062	-0.003
P value	0.904	0.338	0.888	0.224	0.071	0.931
LF/HF	0.015	0.034	0.006	-0.051	-0.064	-0.001
P value	0.660	0.316	0.855	0.142	0.063	0.986
SD1 (ms)	-0.016	-0.073^*^	0.162^**^	0.313^**^	0.259	0.200^**^
P value	0.636	0.035	<0.001	<0.001	<0.001	<0.001
SD2 (ms)	0.024	-0.080^*^	0.202^**^	0.348^*^	0.278^**^	0.242^**^
P value	0.480	0.023	<0.001	<0.001	<0.001	<0.001
SD1- SD2	-0.057	-0.028	0.063	0.117^**^	0.106^*^	0.079^*^
P value	0.100	0.415	0.067	<0.001	0.002	0.022
Alpha1	0.065	0.051	-0.019	-0.068	-0.087^*^	-0.030
P value	0.059	0.151	0.575	0.050	0.011	0.338
Alpha2	-0.022	0.017	-0.075^*^	-0.124^**^	-0.034	-0.086^*^
P value	0.525	0.620	0.030	<0.001	0.326	0.012

P<0.05; ^**^P<0.0001. SDRR: standard deviation of normal RR intervals; RMSSD: root mean square of the RR intervals; pNN50: percentage of normal RR intervals that differ more than 50ms from its adjacent; HF: high-frequency power; LF: low-frequency power; SD1: instantaneous variability of RR intervals; SD2: long-term variability.

## Discussion

In the present study, we aimed to investigate the relationship between HRV indices and age and sex, while adjusting for the confounding effects of the PADL level. Men and women were found to exhibit significant differences in autonomic modulation, even after correction for the PADL level. Moreover, the intensity of PADL was related to certain HRV indices.

We observed that the HRV indices changed as the intensity of PADL increased. Moreover, MVPA showed a positive correlation with certain indices of the parasympathetic nervous system. Such a change in cardiac autonomic modulation may be explained by an improvement in cardiovagal baroreceptor sensitivity and in the central regulation of vagal output, which would increase with physical activity (5). Moreover, the importance of physical activity in autonomic modulation has been well described thus far, as vagal predominance is favored at rest (3,5,12). Another study (5) has described the relationship between moderate to vigorous physical activity and increased HRV; however, in that study, physical activity was assessed using a questionnaire, which is known to have limitations regarding the reliability of the responses.

We found that age and sex were important determinants of HRV indices, consistent with that reported in the literature (3,13,14). Older individuals had lower values of representative indices of the parasympathetic nervous system. With an increase in age, the HRV tends to decrease; this reduction can primarily be observed from the indices in the time domain (13,15). Women exhibited higher values of representative indices of the parasympathetic nervous system, whereas men showed sympathetic predominance in heart modulation, as previously described (3,14–16). Also, HRV strongly declined with age and was consistently higher in women. These demographic factors together explained 17.4 to 21.9% in a previous study, while adding lifestyle and psychosocial factors to the model additionally explained less than 0.50% of the variance in HRV (17). Studies have suggested that female hormones, particularly β-estradiol, are involved in the facilitation of vagal activation in the heart (3). Furthermore, the physical constitution of men would be one possible explanation for the sympathetic predominance; men have increased muscle sympathetic activity in addition to a greater number of sympathetic ganglia compared to women (3).

The selection of BMI as an independent predictor of certain HRV indices can be explained by the relationship between obesity and cardiac autonomic modulation. Studies have shown that HRV is reduced in obese individuals (13,18). Moreover, the correlation between obesity levels and HRV was found to be reduced, and obesity was found to have an effect on the cardiovascular system even before the age at which the CVD risk is high (19). In fact, obese individuals have sympathovagal imbalance that is characterized by increased sympathetic activity and reduced parasympathetic activity in the heart (19), and are hence predisposed to an increased risk for CVD.

The present findings support the results cited in the literature regarding the influence of age and sex on HRV, although the confounding effect of cardiovascular risk factors (particularly physical inactivity) was rarely adjusted for (17). In addition, we emphasize the importance of physical activity in the improvement of cardiac autonomic modulation, as well as the effects of different levels of PADL (objectively evaluated using triaxial accelerometry) on the HRV indices.

It is noteworthy, however, that LF band, as derived from the RR-interval power spectrum, is not related only to the sympathetic branch of the autonomic nervous system but contains information about both sympathetic and parasympathetic activity (15,20). The LF band (0.04–0.15 Hz), measured at a given time, has been considered to be mainly of baroreceptor activity at rest and not a cardiac sympathetic innervation (21). During slow respiration, vagal activity can generate oscillations in the heart rhythms that cross in the LF range. Therefore, vagal influences are particularly present in the LF range (15).

The relationship between LF and HF (LF/HF ratio), in turn, was based on the hypothesis that LF energy can be generated at the same time as HF. Therefore, the LF/HF ratio found in sympathetic or parasympathetic dominance depends on this relationship. However, this supposed sympathetic-vagal balance was widely contested (15), since the LF index is not purely representative of the sympathetic system and also represents the activity of the parasympathetic nervous system, in addition to being more marked by nonspecific factors. Finally, the nonlinear character of the interaction between sympathetic and parasympathetic systems, the influence of measure conditions, and the confounding interaction between breath and heart rate at rest compromise the interpretation of LF/HF as a measure of sympathovagal balance.

The present study has certain limitations. First, due to the use of a convenience sample, a larger proportion of women were enrolled. However, we believe that this bias did not influence the statistical power of the analysis. Second, cardiovascular risk was assessed through a self-reporting method, which could underestimate the true value. Nevertheless, the findings of the present study were similar to those of previous direct evaluations, thus indicating the validity of the reported results. Also, the inclusion of other measurements, as the one derived from blood pressure recordings, or the use of nonlinear indices of HRV, could give important complementary information with respect to the traditional indices assessed as previously done (22).

We can conclude that age and sex are significant predictors of differences in HRV, independent of the objectively measured PADL level in asymptomatic adults. A high level of PADL was associated with moderately better autonomic modulation. Based on our results, we suggest that strategies for preventing autonomic modulation changes in adults be designed while specifically considering sex and age.

## Supplementary Material

Click here to view [pdf].
